# Automated detection and counting of redbanded stink bugs in soybean using an improved computer vision model

**DOI:** 10.3389/frai.2026.1875232

**Published:** 2026-08-14

**Authors:** Saurav Upadhyaya, Jeffrey A. Davis, Ivan Grijalva

**Affiliations:** Department of Entomology, Louisiana State University, Baton Rouge, LA, United States

**Keywords:** counting, detection, redbanded stink bug, soybean, YOLOV8m

## Abstract

The redbanded stink bug (RBSB), *Piezodorus guildinii*, is a major economic pest of soybean, with feeding damage that leads to significant yield losses and increased reliance on pesticide applications. Current management practices depend on manual identification and repeated field counting, which are labor-intensive, time-consuming, and prone to human error, particularly across large production areas. To address these limitations, this study evaluated the potential of computer vision models to automatically detect and count RBSB adults using imagery. A total of 2,281 field images were collected under varying stink bug densities using different sensors. These images were used to train multiple YOLOv8 model variants for automated detection and counting. The best-performing model was further enhanced by integrating a convolutional block attention module (CBAM) and adaptive spatial feature fusion (ASFF) to improve detection accuracy and counting performance, which are critical for informed pest management decisions. The improved YOLOv8m model achieved 96.30% precision, 76.40% recall, F1-score of 85.20, 82.00% mean average precision (mAP50), and 75.30% mean average precision (mAP50-95) for detecting and counting RBSB adults in images, outperforming the baseline model, which showed lower performance with 95.68% precision, 76.69% recall, F1-score of 85.13, 78.70% mAP50, and 66.00% mAP50-95. The enhanced model was deployed in a prototype web application to evaluate its practical capabilities. This application allows users to upload images and visualizes detection bounding boxes and RBSB counts, resulting in low misdetection error. Overall, the framework and results presented in this study provide a practical alternative to manual identification of RBSB adults in soybean systems using images and have the potential to improve traditional pest monitoring practices.

## 1 Introduction

Soybeans are an economically important cash crop with an export value of approximately 24 billion in the United States in 2024 (Colussi and Langemeier, 2025). Between 2005 and 2007, global soybean production increased by 4.6% per year, reaching an average of 217.6 million tons (Masuda and Goldsmith, 2009). It is expected to reach 371.3 million tons by 2030, growing at 2.2% annually (Masuda and Goldsmith, 2009). However, soybean production has been negatively affected by stink bugs that feed on various parts of the soybean plant, resulting in reduced yields (Michel et al., 2015). These closely related species share very similar morphological traits, which makes visual identification and counting difficult; examples include *Piezodorus guildinii* (Westwood, 1837) (redbanded stink bug, RBSB) and *Thyanta custator* (Fabricius, 1775) (red-shouldered stink bug) (Hernowo et al., 2020).

Among the various species of stink bugs, RBSB is considered one of the most economically important and frequently encountered pests during soybean field scouting in the Midsouth soybean fields (Michel et al., 2015; Bastola, 2017). This species causes significant damage to leaves, stems, flowers, and developing pods (des Bordes et al., 2026). The pest feeds by inserting its stylets into soybean pods and injecting toxic salivary enzymes through the pod wall upon locating developing seeds (Bastola, 2017; Okosun et al., 2024). These enzymes degrade plant tissues into a digestible liquid, which the insects then consume, leading to seed damage and yield reduction (Bastola, 2017; Okosun et al., 2024). In addition to yield loss, it causes visible seed damage, such as discoloration, wrinkling or shriveling, dark puncture wounds, and scars on the seed coat, all of which lower seed quality and market value (Corrêa-Ferreira and De Azevedo, 2002).

The management of this species typically relies on pesticide applications guided by action thresholds after scouting (Leach et al., 2025). Scouting commonly involves the use of sweep nets to collect adult insects across transects in soybean fields, combined with visual identification and counting to estimate population density (Davis, 2022). However, this approach can be time-consuming when large soybean acreages must be sampled, prone to errors, especially when closely related species share similar morphological traits, and inefficient because it depends on visual observations. As a result computer vision models are increasingly being used to automate pest identification and counting for pest management decisions (Chithambarathanu and Jeyakumar, 2023). In recent years, several advanced computer vision models, such as YOLO, Mask R-CNN, and Faster R-CNN with architectural enhancements, have been applied to automate pest identification.

For example, an enhanced Cascade R-CNN PH ResNet50 architecture was developed and evaluated against YOLOv5, YOLOv6, YOLOv7, YOLOv8, Faster R-CNN, and Cascade R-CNN models for monitoring ten planthopper species. The enhanced model achieved an average recall of 83.43% and an average precision of 83.56% (Sheng et al., 2024). Similarly, different YOLO models were used detect and identify populations of *Laodelphax striatellus* (Fallén, 1826) (the small brown planthopper). By integrating a convolutional block attention module (CBAM) and adaptive spatial feature fusion (ASFF) into YOLOv5s, they increased the mean average precision (mAP50) from 90 to 94% (Huang et al., 2023). The integration of CBAM helped supress background noise and redirect the model’s attention toward pest regions, compared to the original model (Woo et al., 2018), while ASFF merge features across different scales into a unified representation, thereby improving detection performance (Liu et al., 2019).

Comparable model improvements have been reported in other pest detection studies. In one study, a modified YOLOv5s model was used to detect and count *Myzus persicae* (Sulzer, 1776; green peach aphid). Their approach enhanced feature representation by integrating a convolutional block attention module (CBAM) into the backbone and replacing PANet with a bidirectional feature pyramid network, resulting in a substantial increase in mAP50 from 87.30 to 99.30% (Li et al., 2023). Similarly, incorporating CBAM into a YOLOv8 model improved the detection and quantification of blurred and small-object pests, such as rice planthoppers, achieving an increase in mAP50 from 69.20 to 88.6% (Wang et al., 2025). More recently, attention-based detectors such as RT-DETR have shown strong performance in complex scenes, particularly for occluded and densely clustered pests (Mafuwe et al., 2026), however, this is less critical for RBSB adults, which are typically more dispersed across the field (Okosun et al., 2024).

**Figure 1:**
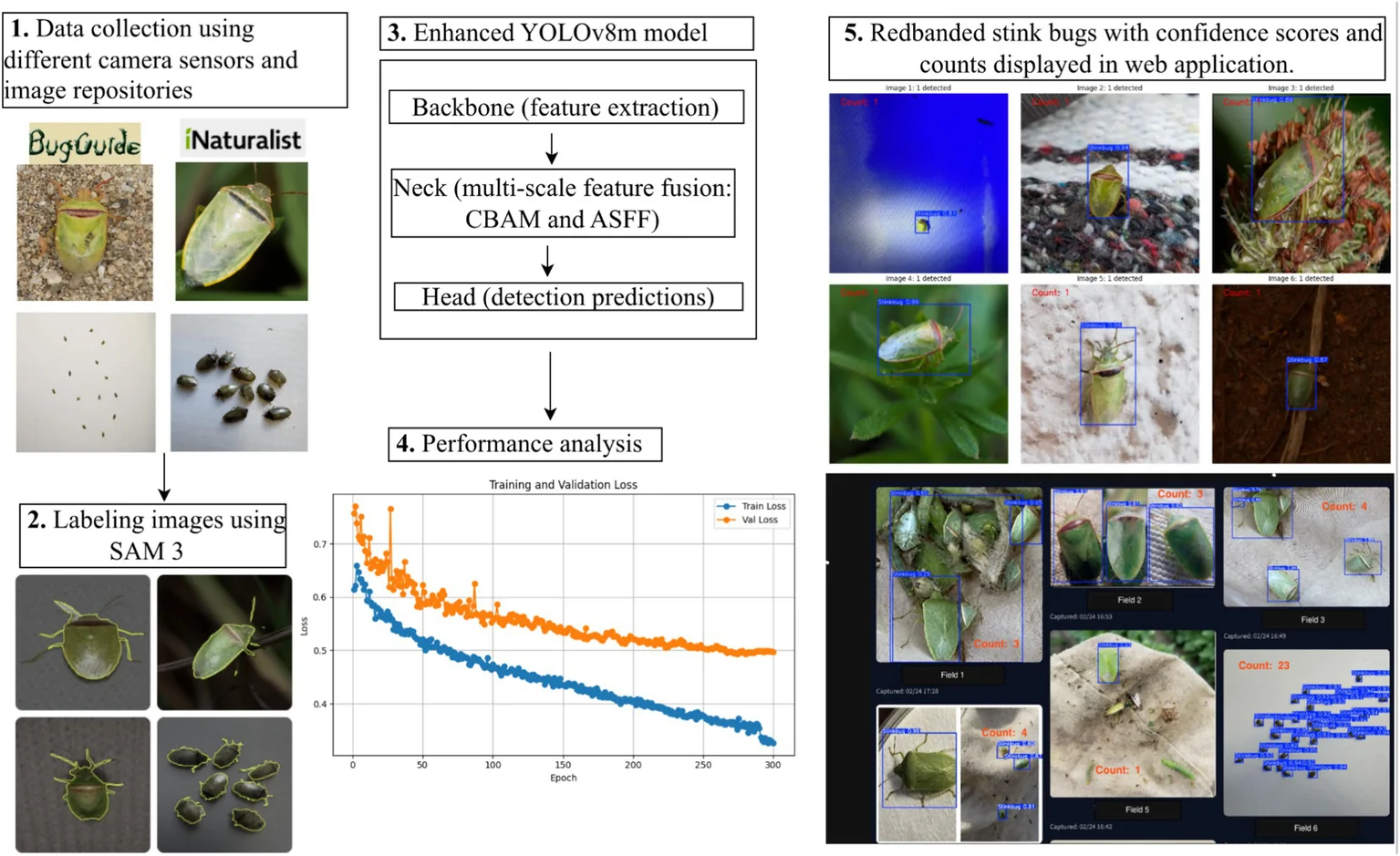
Framework to detect and count RBSB adults in images using computer vision models.

Despite recent advances in computer vision for pest detection, no enhanced model has been specifically developed to accurately detect and count RBSB adults to support soybean pest monitoring, which still relies heavily on visual observations. To address this gap, this study developed an improved computer vision model that automatically detects and counts RBSB adults from uploaded images. The model reduces background noise across diverse image conditions, commonly found in field settings, and improves detection accuracy across varying object sizes (i.e., RBSB adults) through the integration of CBAM + ASFF enhanced modules. The model was integrated into a prototype application that enables users to upload images and obtain reliable RBSB detections and counts in real-time as an initial decision support tool that needs further field validation. Overall, the proposed framework (Figure 1) offers a practical alternative to manual pest identification and has the potential to improve pest monitoring efficiency—defined as the process of tracking and evaluating pest populations—while reducing reliance on subjective visual observations.

## 2 Materials and methods

### 2.1 Dataset collection

RBSB adults were obtained from established rearing colonies housed in the Life Sciences Building, Department of Biological Sciences, Louisiana State University, Baton Rouge, 70,803. These colonies are maintained for continuous growth to ensure a steady supply of specimens.

RBSB populations can exhibit temporal and spatial variability within soybean fields (Bastola, 2017), which can limit consistent collection of species-specific imagery. To address these limitations, we created a custom dataset of 2,281 images, each containing 1 to 25 RBSB adults, including 2,010 images from established rearing colonies and 271 field images sourced from iNaturalist and BugGuide imagery repositories. Images were captured with a Nikon Coolpix P600 and a NIKKOR Z 24–70 mm f/4 S lens, and an iPhone 15 Pro Max, all mounted perpendicularly to the target plane. This standardized setup was designed to guide end users in capturing images to achieve optimal performance with the enhanced model.

**Figure 2:**
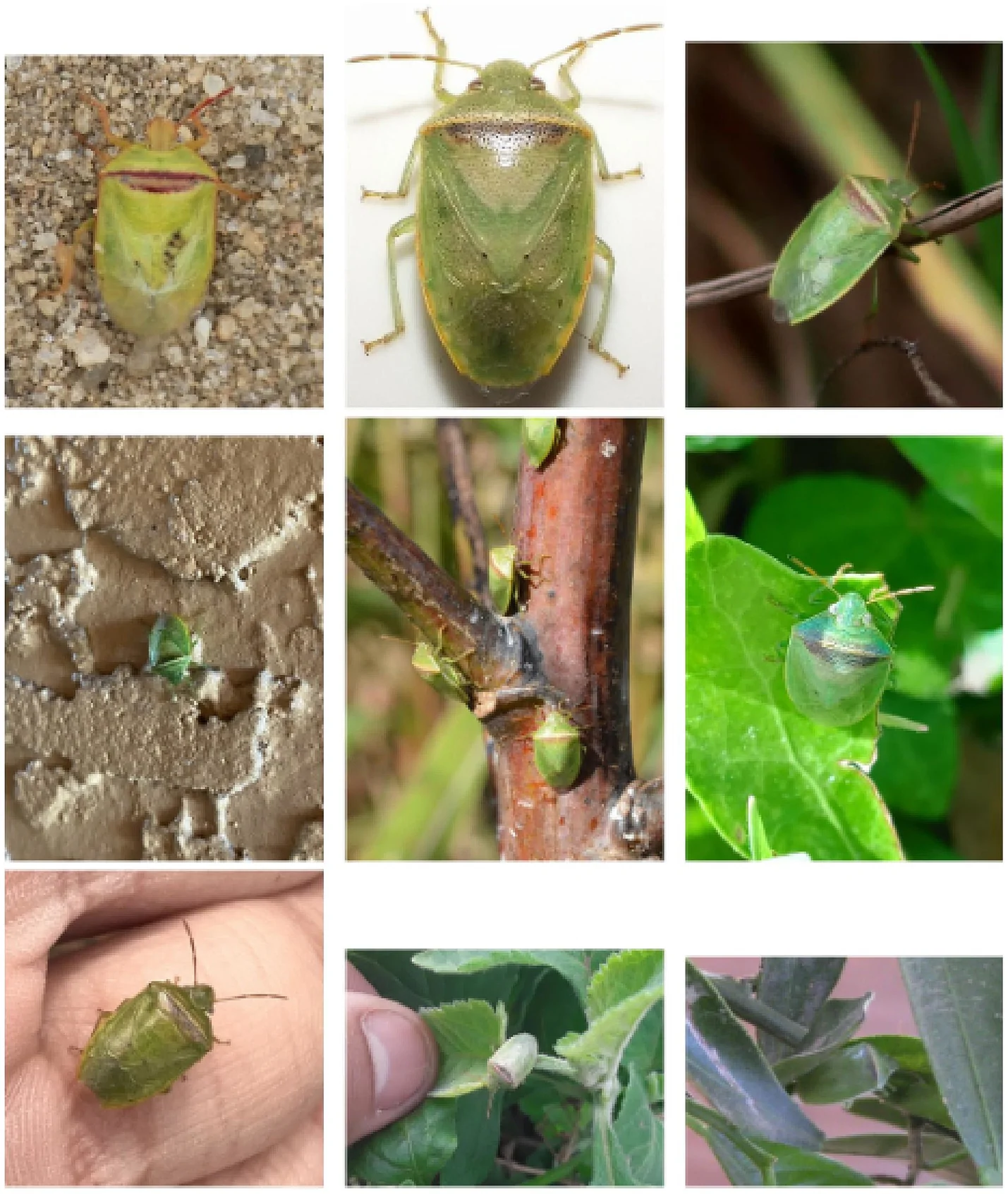
Images with RBSB adults present retrieved from iNaturalist and BugGuide repositories.

Camera angles were fixed top-down, and specimens were randomly placed on a flat surface under different lighting conditions (3,500, 4,500, 5,500) measured in Kelvin (unit of color temperature), and natural daylight to simulate field-relevant illumination. The imaging parameters included a sensor-to-target distance of 60 mm, a focal length of 50 mm, an aperture of f/22, autofocus (AF-F), and an exposure time of 0.4 s. The images were acquired in standard red green and blue format (RGB) of 6,048 × 4,024 pixels for model training. To enhance dataset diversity, we also included field images from iNaturalist and BugGuide (Figure 2), publicly available platforms that host images of insects, including RBSB adults. The combination of multiple imaging sources and sensors improves dataset representativeness and supports robust model training and evaluation.

### 2.2 Dataset labelling

**Figure 3:**
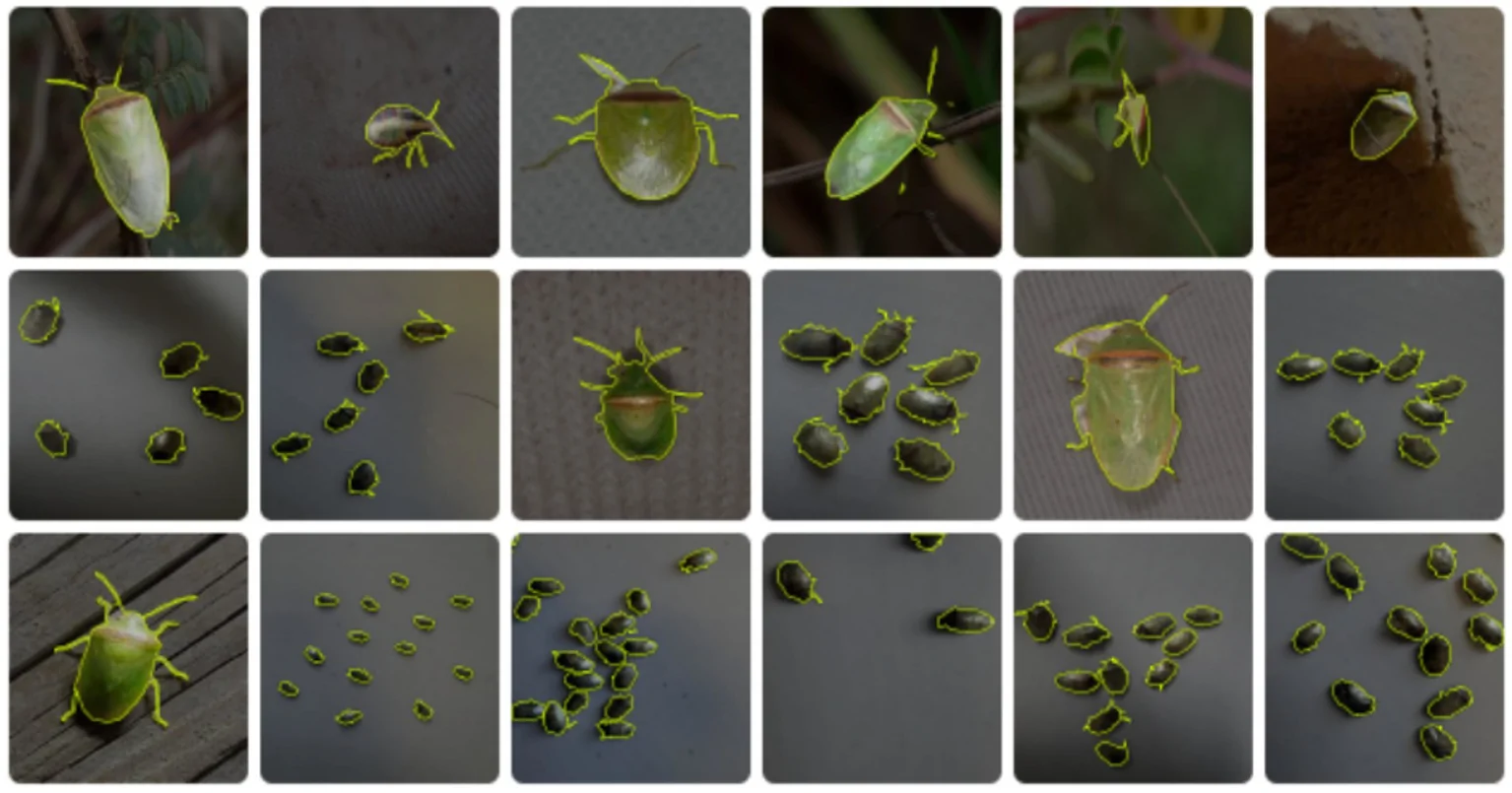
Labeled dataset of RBSB adults for model training.

For data annotation, we used Roboflow, a cloud-based platform designed for labelling, preprocessing, training, and deploying computer vision models (Dwyer et al., 2024). Our annotation pipeline began with uploading a dataset of 2,281 collected images, each containing 1–25 RBSB adults. Using Roboflow’s integrated SAM 3 Model, we labeled every RBSB adult within each image. SAM 3, supports labelling through promptable concept segmentation for both images and videos (Carion et al., 2025). The model can detect, segment, and track all occurrences of an object guided by text prompts, making it valuable for labelling. To leverage this labelling feature, we used the SAM 3 model within the platform, the model generated 29,292 segmentation-format annotations, which were reviewed after each label performed to ensure reliable labeling for training dataset (Figure 3).

### 2.3 Model selection

YOLO models are widely preferred for detection tasks due to their speed and accuracy (Vijayakumar and Vairavasundaram, 2024). Leveraging a one-stage detector architecture, YOLO models simultaneously predict bounding boxes and class probabilities within a single pass, resulting in exceptionally low inference times (Ahmad et al., 2020). This efficiency is particularly beneficial for rapid inference and object counting applications, such as pest detection (Likith et al., 2021; Padma Priya et al., 2024; Grijalva et al., 2025; Hidayatullah et al., 2025).

YOLOv8, one of the different versions in the YOLO family, introduces several enhancements over previous versions, enabling advanced tasks such as segmentation, classification, and tracking, as well as improved small-object detection. Notably, YOLOv8 incorporates new data augmentation strategies, CutMix and Mixup (Varghese and Sambath, 2024; Hidayatullah et al., 2025), to further boost object detection accuracy. Its anchor-free detection head allows independent processing of detection and classification (Varghese and Sambath, 2024). Given their advanced features and proven effectiveness in insect detection, YOLOv8 and its variants (small (s), medium (m), large (l), and extra-large (x) were selected for the detection and counting of RBSB adults. Futhermore, the best performed model was enhanced with modules to increase detection and counting accuracy.

### 2.4 Dataset training, hyperparameters, and computer software

We partitioned the dataset of 2,281 images into training, validation, and test sets at a 70:15:15 ratio. Specifically, 70% of images were allocated to the training set, while 15% each was reserved for validation and testing. Prior to training, all images were resized to a standard dimension of pixels (640 × 640 pixels) to suit the requirements of the YOLOv8 variants (small (s), medium (m), large (l), and extra large (x) (Sapkota et al., 2024). Experiments were performed on an NVIDIA GPU (CUDA 12.8 version), using the following hyperparameter settings: 1,000 training epochs, a batch size of 32, a patience value of 10 (early stopping was initiated if no improvement was observed over 10 consecutive validation checks), and two workers enabling parallel data loading to accelerate training. The models evaluated under these conditions included YOLOv8s, YOLOv8m, YOLOv8l, and YOLOv8x. Computational experiments were performed using a Dell Pro Max Tower T2 FCT 2250 with an NVIDIA RTX 4000 Ada Generation (Windows 11 Pro), 64 GB RAM, 24 Cores, 20 GB Graphics Card, Intel (R) Core (TM) Ultra 92,895, and 1.86 TB storage. Labeling, training, and validation analyses were performed using Roboflow, Visual Studio Code, Jupyter Notebook, CUDA (12.8), PyTorch (2.7), and Python (3.12.8).

### 2.5 Model enhancement

**Figure 4:**
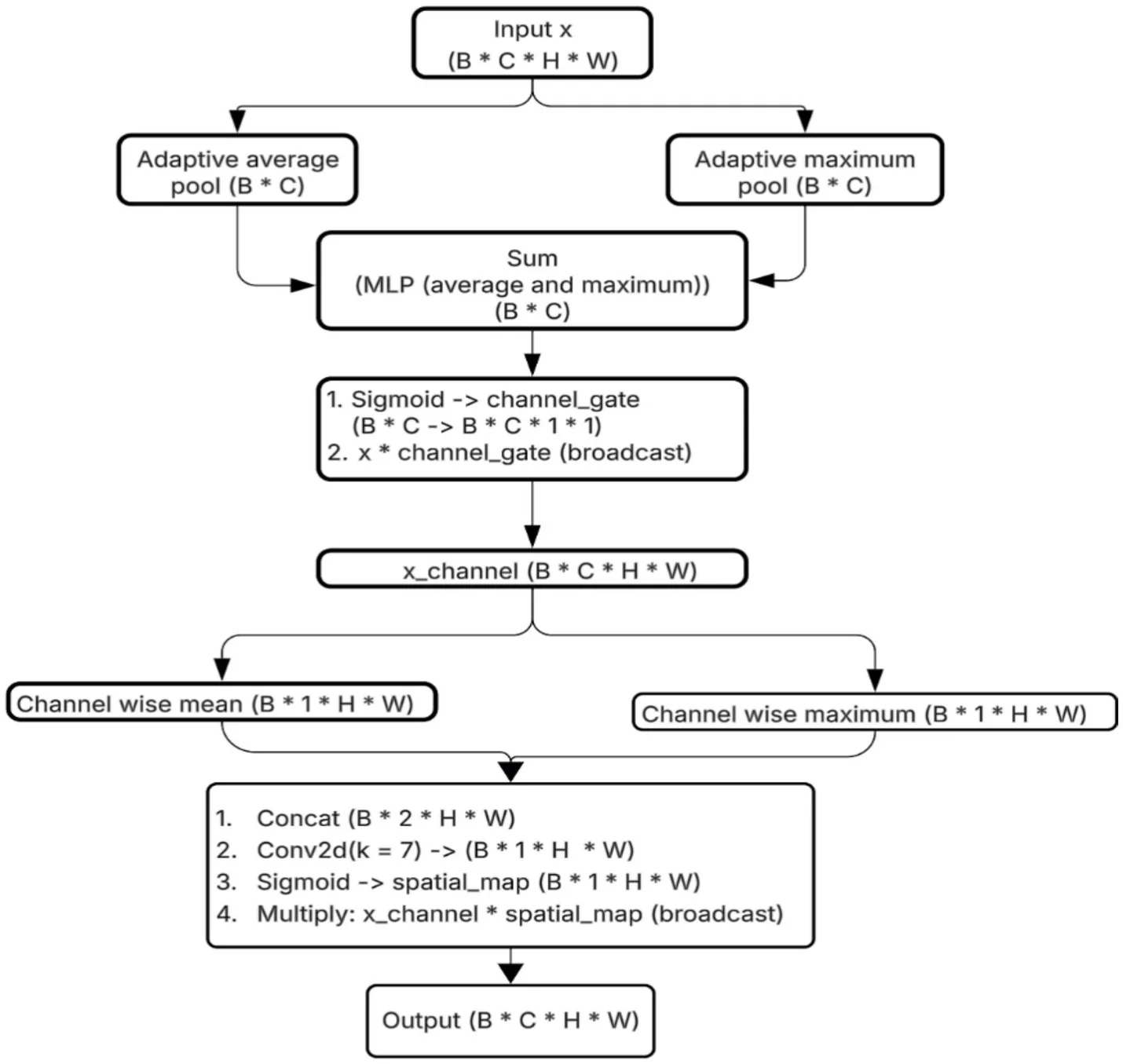
Structure of CBAM used to enhance YOLOv8m model.

**Figure 5:**
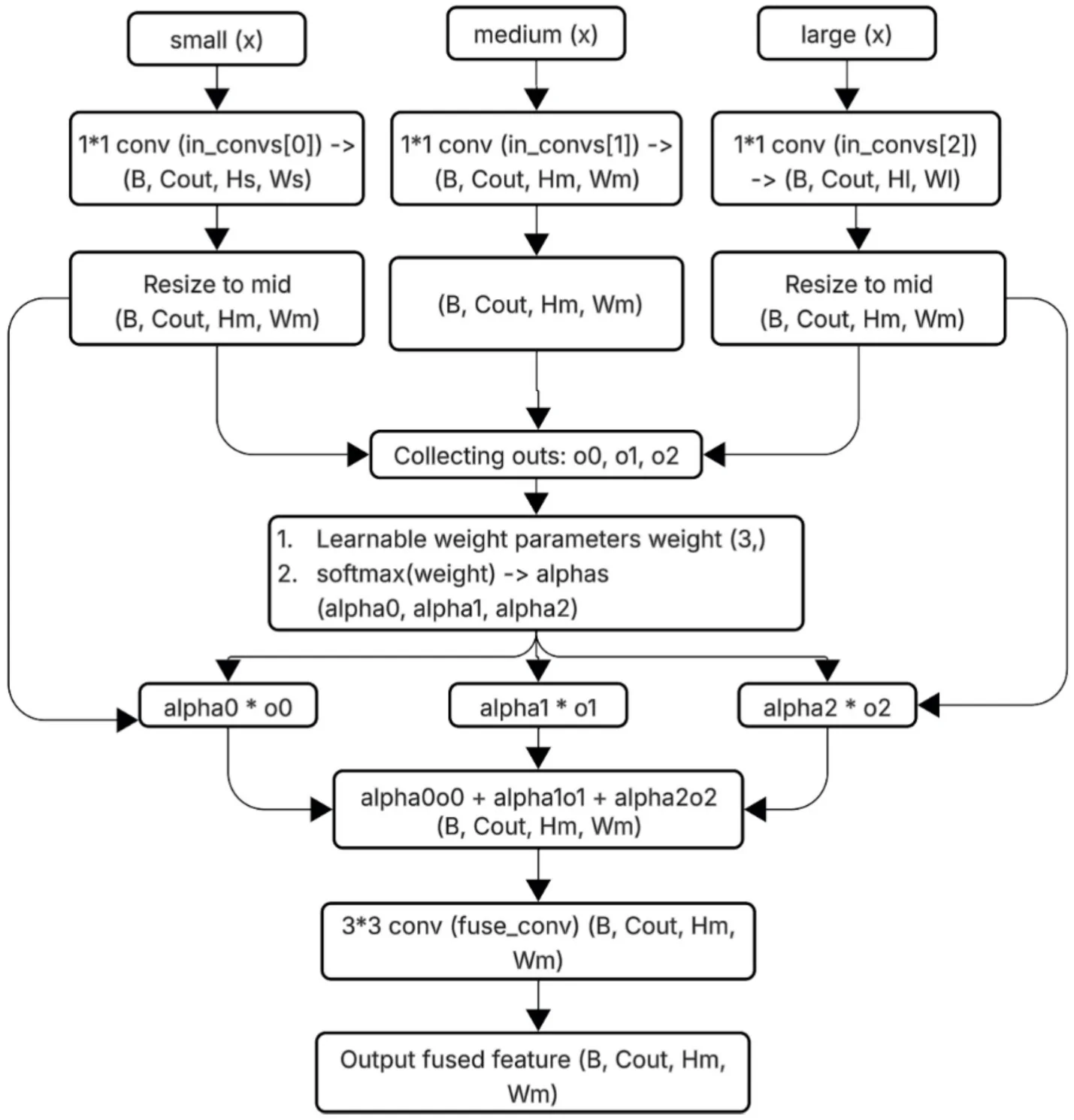
Structure of ASFF used to enhance YOLOv8m model.

To further enhance detection and counting performance, we integrated the Convolutional Block Attention Module (CBAM) (Figure 4) and Adaptive Spatial Feature Fusion (ASFF) (Figure 5) into the YOLOv8m model architecture due to its greater capacity for enhancement and their applicability in pest monitoring contexts (Pan and Daoliang, 2022; Sapkota et al., 2024; Vijayakumar and Vairavasundaram, 2024; Cui et al., 2025; Hidayatullah et al., 2025). The CBAM and ASFF modules were integrated to improve the model’s ability to focus on the boundaries of RBSB adults and accurately localize them in images while reducing the influence of background noise. In addition, because the dataset contained RBSB adults at different sizes, ASFF was incorporated to effectively learn features across multiple object scales and preserve the most informative scale-specific features at each pixel. This combination delivered a favorable trade-off between detection accuracy and inference speed, improving the model’s focus on relevant features and its fusion of multi-scale information.

The CBAM module was applied to mid-level features, concentrating on critical elements such as edges and outlines of RBSB adults while attenuating background noise. Specifically, CBAM uses channel- and spatial-attention mechanisms to highlight important spatial and channel-wise characteristics in RBSB adult images (Woo et al., 2018; Pan and Daoliang, 2022). Adaptive average pooling captured global spatial information (e.g., background color), while adaptive maximum pooling emphasized localized features such as RBSB adult outlines. Through a multi-layer perceptron, channels that best identified the shape of RBSB adults were prioritized. The resulting attention scores were refined via a sigmoid activation (“channel gate”), amplifying salient features. Post-channel gating, the model focused more on relevant regions, improving the detection of RBSB adults. The spatial attention mechanism further refined these outputs, generating heatmaps and enabling precise counting of RBSB adults.

**Figure 6:**
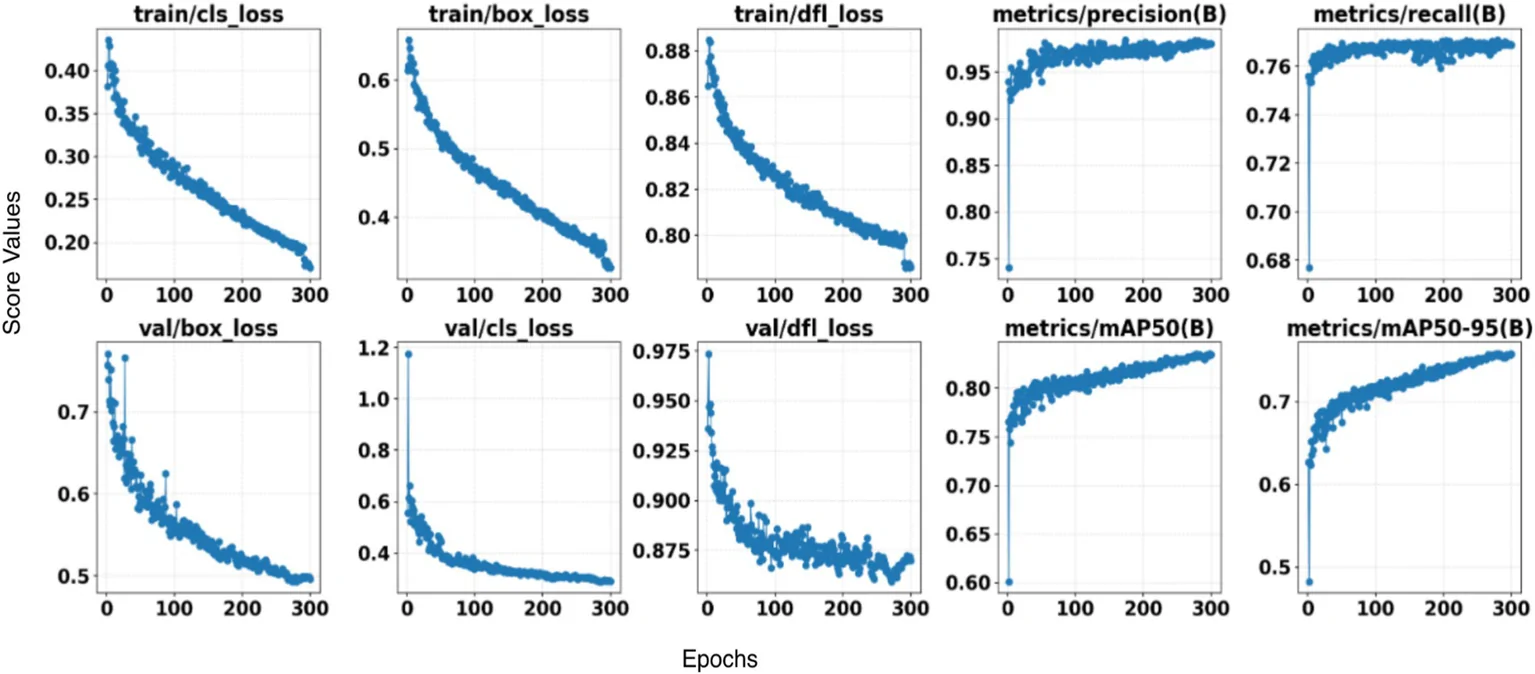
Learning curves of loss, precision, and recall metrics based on YOLOv8m (CBAM + ASFF) model.

In addition, ASFF was used to merge features across different scales (small, mid, and large) into a unified representation, facilitating the detection of RBSB adults regardless of their size. The overall architecture positions the CBAM and ASFF modules within the backbone and neck regions of YOLOv8m. The backbone extracts multi-scale features, which are processed by CBAM for attention refinement and subsequently fused by ASFF. The neck, utilizing a Path Aggregation Network, aggregates these features before passing them to the head for final prediction (Zhang et al., 2023; Cui et al., 2025). During training, the model’s predictions are evaluated against ground-truth annotations using metrics such as box loss, class loss, and objectness (Figure 6). These losses guide backpropagation, enabling the model to iteratively improve its localization and classification capabilities. Given its superior performance compared to other YOLOv8 variants, the YOLOv8m model enhanced with CBAM and ASFF was ultimately chosen and deployed in a web application for the detection and counting of RBSB adults.

### 2.6 Metrics of performance

This study used various standard evaluation metrics to measure the performance of different YOLO models (Liu and Huang, 2018; Gündüz and Işık, 2023). These metrics help determine the most effective model for detecting and counting RBSB adults in images. The metrics used included:

#### 2.6.1 Object confidence

It is a metric that assesses how reliably the YOLOv8 (s/m/l/x) models indicate that a predicted bounding box actually contains the target object (Chengji et al., 2018). It was used to determine whether RBSB adults were present in the images. Mathematically, it is represented as

$$\mathrm{Conf}\left(\mathrm{Object}\right)=\mathrm{Pr}\left(\mathrm{Object}\right)*{\mathrm{IOU}}_{\mathrm{Pred}}^{\mathrm{Truth}}\;$$

Where Pr(Object) belongs to the set {0,1} the Intersection over Union (IOU) is.

represented as:


$${\mathrm{IOU}}_{Pred}^{Truth}=\frac{\mathrm{area}\left(\mathrm{box}\left(\mathrm{Truth}\right)�\mathrm{box}\left(\mathrm{Pred}\right)\right)}{area\left(box\left(Truth\right)�box\left(Pred\right)\right)}$$

#### 2.6.2 Precision

It is a metric that measures the model’s accuracy in positive predictions (Ajayi et al., 2023). Specifically, it indicates the proportion of RBSB adults detected by the model that are truly correct among all identified adults. Thus, Precision quantifies the percentage of positive predictions that are truly correct. It is defined as the ratio of true positives (TP) to the sum of true positives and false positives (TP + FP), where FP denotes false positives. It is represented as:


$$Precision\;\left(P\right)=\frac{TruePositive\;\left(TP\right)}{TruePositive\;\left(TP\right)+FalsePositive\;\left(FP\right)}$$

#### 2.6.3 Recall

It is a metric that measures how well the model identifies all relevant objects in our dataset (Ajayi et al., 2023). In our study, recall evaluates how well the model identifies all actual RBSB adults in an image. It reflects the proportion of true RBSB adults that the model successfully detects out of the total number present. It is represented as:


$$Recall\;\left(R\right)=\frac{TruePositive\;\left(TP\right)}{TruePositive\;\left(TP\right)+FalseNegative\;\left(FN\right)}$$

TP refers to the number of times the model accurately detected RBSB adults, while FP indicates instances where the model incorrectly label as RBSB adults. The sum TP + FP represents the total number of identifications made by the model.

#### 2.6.4 F1-score

It is a performance metric that balances precision and recall, giving a single measure of detection accuracy. A high F1-score indicates that automated RBSB counts match manual counts closely (Rainio et al., 2024).


$$F_{1}=2*\frac{Precision*Recall}{Precision+Recall}$$

#### 2.6.5 Mean absolute error

It is a measurement that assesses different models’ prediction accuracy by determining the average magnitude of the errors (Robeson and Willmott, 2023). In this study, it was used to quantify the average difference between the predicted number of RBSB adults and the actual count (ground truth) across all datasets. It is represented as:


$$MAE=\frac{1}{N}\;\sum_{i=1}^{N}\left|y_{i}-\hat{y_{i}}\right|\;$$

Where N is the total number of RBSB adults, y_i_ is the ground truth, ( ${\hat{y}}_{i}$ ) is the prediction.

Mean average precision (mAP): It is a metric that summarizes how well a model detects objects across all classes and confidence thresholds (50, 50–95) by combining precision and recall into a single score (Gündüz and Işık, 2023). The calculation averages the individual average precision (AP) scores for each object class in the dataset. AP itself evaluates the model’s precision and recall for detecting a specific class. It is represented as:


$$mAP=\frac{\sum_{q=1}^{Q}AveP\left(q\right)}{\mathrm{Precision}+\mathrm{Recall}}$$

### 2.7 Web application development and interface

**Figure 7:**
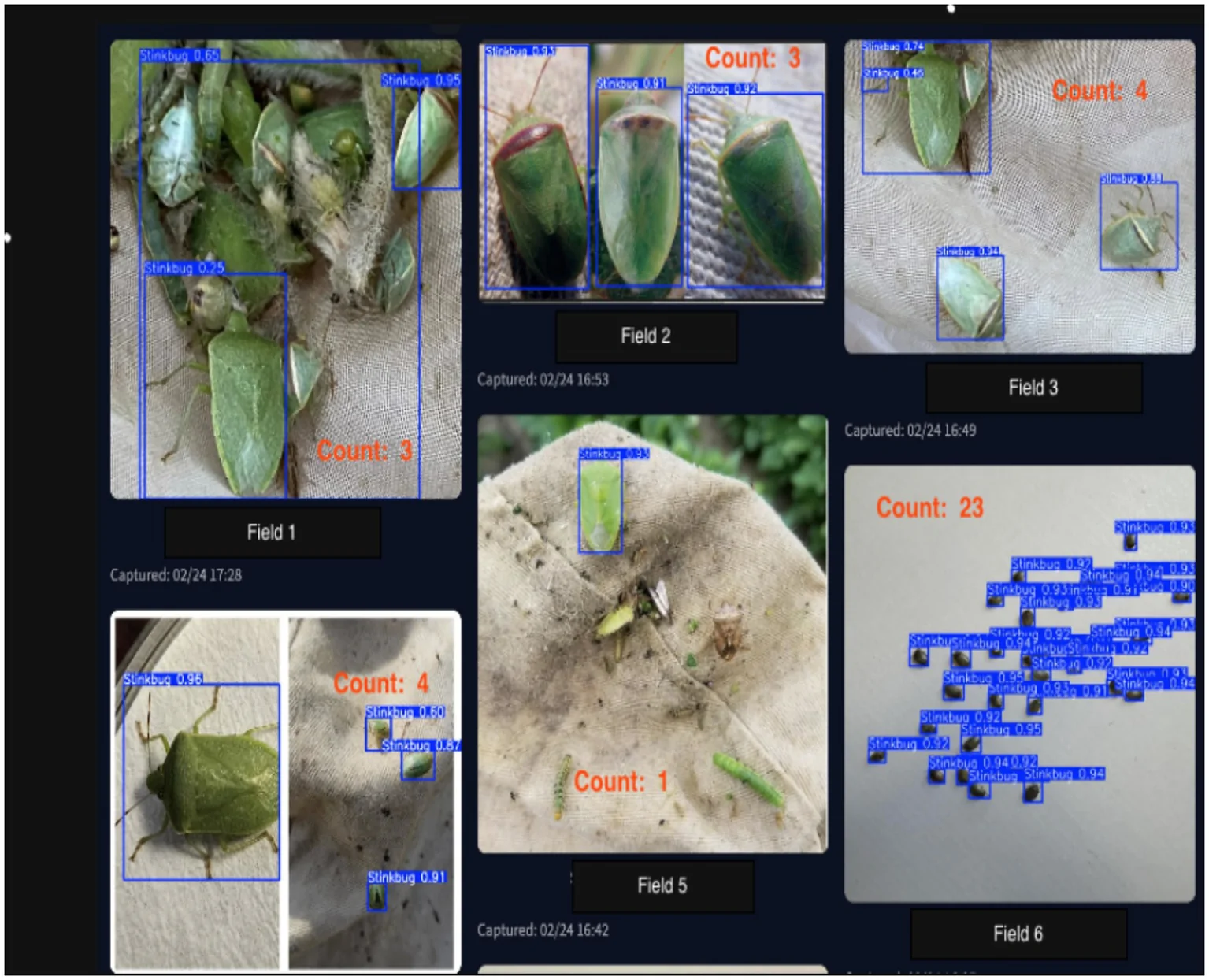
Testing images with detections, counts and misdetections performed by enhanced YOLOV8m model in the web interface.

The main goal of the application was to assess the performance of the enhanced model as an initial decision support tool, which will require further validation under field conditions. The pest detection platform was developed using Streamlit, a Python framework designed for rapid prototyping of interactive, data-driven applications (Khorasani et al., 2025). Streamlit was chosen for its seamless integration with machine learning workflows and for enabling real-time user interaction (Padma Priya et al., 2024). The core inference engine utilizes the enhanced YOLOv8m model to detect and count RBSB adults within the application interface. The web application offers several features, including image capture for pest detection, and counting. Users can upload the images in the application and obtain counts of pest populations. Once an image is submitted, it is processed by the enhanced YOLOv8m model, displaying detection results including bounding boxes, labels, and count annotations directly on the uploaded image (Figure 7). Overall, this web application addresses the applicability of computer vision models by automating detection and providing counts of RBSB compared with traditional approaches.

## 3 Results

### 3.1 Performance evaluation metrics of YOLO models

All evaluated YOLOv8 model variants consistently achieved over 95% precision, mAP50, mAP50-95, and recall on the test dataset, with a low mean absolute error ranging from 3.15 to 3.23 (Table 1). Among them, YOLOv8s demonstrated the highest precision (96%), recall (76.80%), F1-score (85.33%), and mAP50 (79.30%), while also having the fewest architecture parameters (9 million). In comparison, YOLOv8m, with roughly three times more parameters (25 million), achieved similar metrics: 95.68% precision, 76.69% recall, 85.13, and 78.70% mAP50, comparable to YOLOv8l and YOLOv8x, which contain 55 million and 90 million parameters, respectively, while requiring slower inference times. This indicates that YOLOv8m offers an optimal balance between accuracy and computational efficiency, with a fast inference time of 4.6 ms (Table 1).

**Table 1:** Comparison of different YOLO models on the test dataset.

YOLO models	Precision (%)	Recall (%)	F1-score (%)	mAP50 (%)	mAP50-95 (%)	Mean absolute error	Inference time (ms)	Parameters (millions)
YOLOv8s	96.00	76.80	85.33	79.30	67.40	3.20	2.20	9
YOLOv8m	95.68	76.69	85.13	78.70	66.00	3.22	4.60	25
YOLOv8l	95.70	76.70	85.13	78.00	65.50	3.23	7.20	55
YOLOv8x	95.80	76.80	85.25	79.00	67.40	3.18	11.00	90
**YOLOv8m (CBAM + ASFF)**	**96.30**	**76.40**	**85.20**	**82.00**	**75.30**	**3.15**	**15.90**	**25**

The best performed model is bolded.

### 3.2 Performance evaluation metrics of enhanced YOLOv8m model

To further enhance detection and counting accuracy, YOLOv8m was enhanced by integrating CBAM and ASFF modules. The resulting model achieved a mAP50 of 82%, mAP50-95 of 75.30%, and an F1-score of 85.20%, outperforming the standard YOLOv8 variants (Table 1). It maintained a 96.30% precision, a 76.40% recall, and a low mean absolute error of 3.15, although its inference time increased to nearly 16 ms. With a mAP50-95 of 75.30%, the enhanced model outperformed every other model variant, including the much larger YOLOv8x, which achieved 67.40%. Similarly, the comparison between the baseline YOLOv8m model and the enhanced YOLOv8m integrated with CBAM and ASFF demonstrates that emphasizing relevant features, such as the edges of RBSB adults, while reducing background interference improved precision from 95.68 to 96.30%.

**Figure 8:**
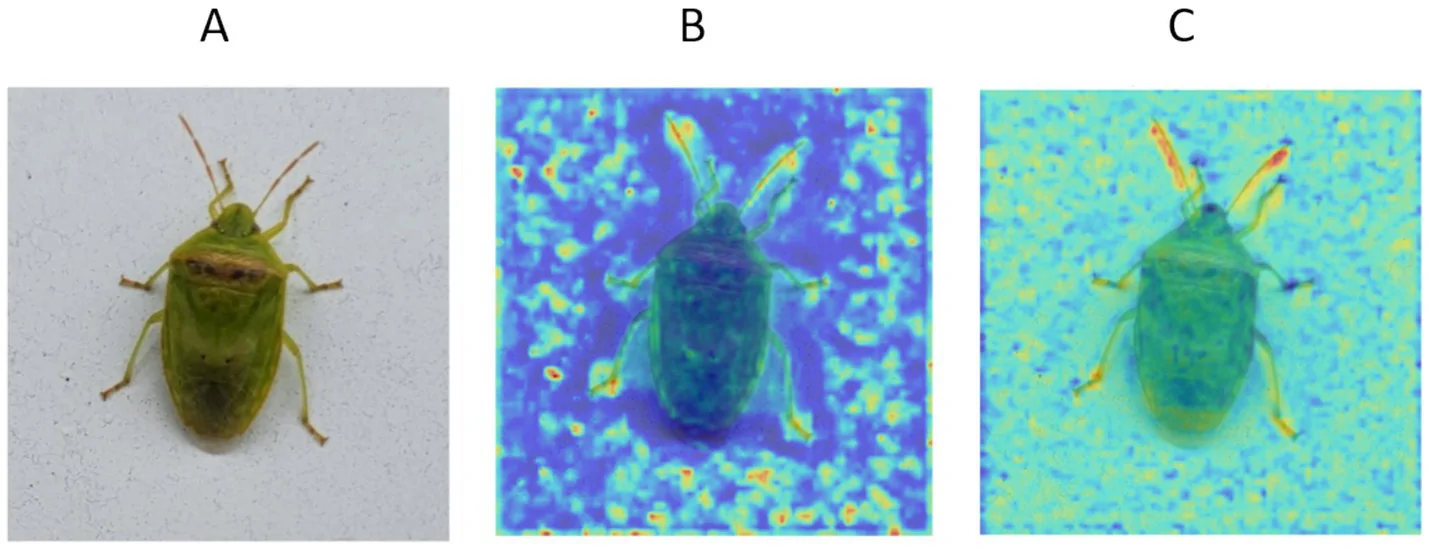
Comparison of feature activation maps into the YOLOv8m framework. Original image **(A)**, YOLOv8m **(B)**, YOLOv8m (CBAM + ASFF) **(C)**.

Furthermore, the incorporation of ASFF enabled more effective handling of RBSB adults appearing at different scales, resulting in an increase in mAP50-95 from 66.00 to 75.30% (Table 1). The effect of integrating CBAM and ASFF into the YOLOv8m architecture is further illustrated through feature activation map comparisons, showing model attention before and after enhancement (Figure 8).

This demonstrates that the enhanced model is significantly better at detecting RBSB adults, even under strict overlap requirements. However, the inference time of improved model is 5 times more than baseline models because of the integration of CBAM and ASFF, highlighting the trade-off between precision and inference time. The model also prioritizes precise predictions over bounding-box generation, leading to a precision–recall trade-off in which some uncertain RBSB adults may be missed, resulting in reduced recall. Despite this, the integration of CBAM (an attention mechanism) and ASFF (which improves detection across varying object scales) enhances the model’s robustness and enables state-of-the-art performance in detecting and counting RBSB adults.

### 3.3 Enhanced YOLOv8m model validation and deployment

**Figure 9:**
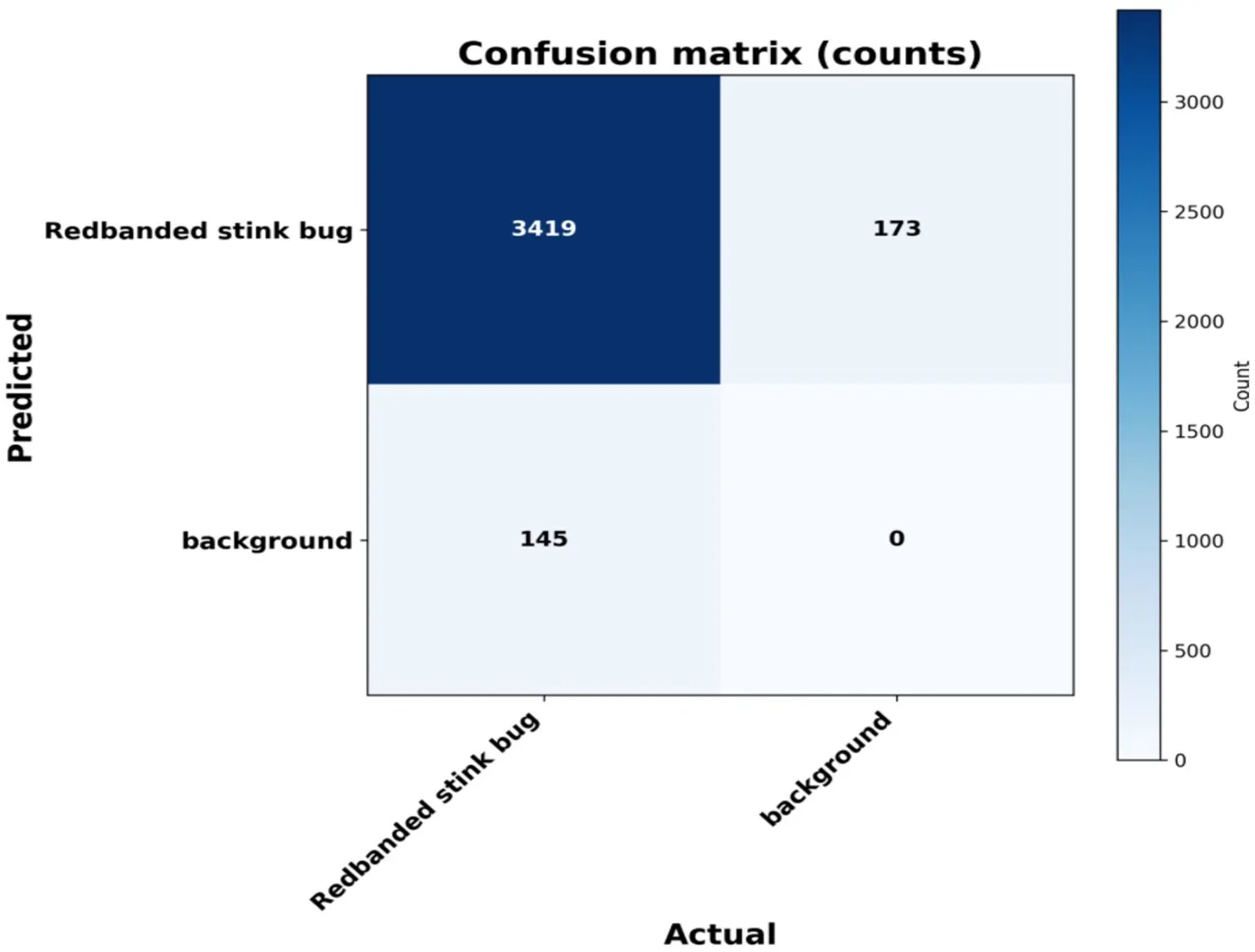
Confusion matrix of the enhanced YOLOv8m model.

**Figure 10:**
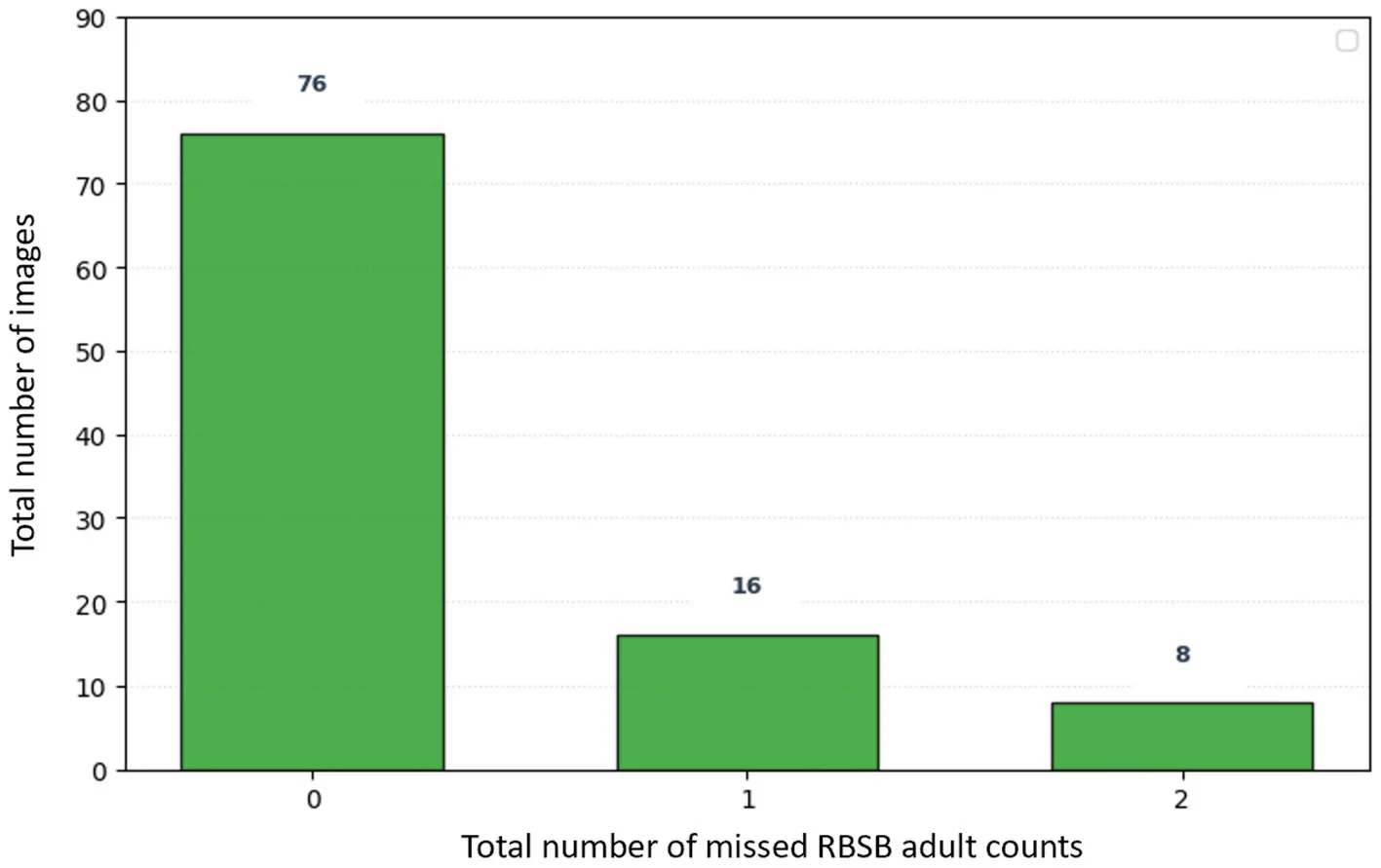
Counting performance of RBSB adults by enhanced model using 100 testing images.

A confusion matrix of the enhanced model was created using our test dataset based on detection counts (Figure 9). Out of a total of 3,737 detections, the enhanced model correctly identified 3,419 stink bugs, corresponding to 91.5% correct detection of RBSB adults, while 318 objects were overcounted as stink bugs, representing an 8.5% misdetection rate on the test dataset. Occasional misdetection occurred due to similarities in color, size, and shape with the backgrounds. Furthemore, we visually counted RBSB adults on 100 unseen images selected randomly and we used the enhanced model for counting inferences. Our model detected and counted RBSB adults accurately in 76 images and only on 24 images missed RBSB adult counts (Figure 10).

Nevertheless, since the enhanced model showed minimal error, it was deployed in a Streamlit application for inference and understand the capabilities of computer vision models in stink bug detection and counting. In the web application, the enhanced model was used for inference. Users can start counting RBSB adults by uploading images using the capture image feature. After uploading, detection results are displayed, showing bounding boxes, counts, and confidence scores for RBSB adults.

## 4 Discussion

### 4.1 Comparison of YOLOv8 model’s performance

Our results show that different YOLOv8 (s/m/l/x) models have varying performance metrics in detecting RBSB adults, primarily due to differences in parameter count and inference speed (Wang and Hao, 2024; Yaseen, 2024). Parameters are critical in YOLO models because the number of parameters determines the model’s complexity to detect objects and influences the deployment performance of a structured model (Diwan et al., 2023). Lighter models, such as YOLOv8s with 9 million parameters, yield faster inference times, while more complex models with over 55 million parameters, such as YOLOv8l and YOLOv8x, tend to be slower, creating a trade-off between efficiency and overall effectiveness (Diwan et al., 2023; Wang and Hao, 2024).

Both YOLOv8s and YOLOv8m showed strong results on the test dataset for detection and counting, achieving nearly the same mean average precision (mAP), but with different inference speeds. In practical terms, both models reliably detect RBSB adults; however, the larger YOLOv8m provides a better foundation for further improvements due to its greater capacity for further enhancement (Diwan et al., 2023; Yaseen, 2024). Our results demonstrate the suitability of YOLOv8m for pest detection, as it offers a good trade-off between computational efficiency and overall effectiveness, consistent with findings reported in related studies (Yue et al., 2024; Grijalva et al., 2025; Abinaya and Daniel, 2026).

### 4.2 Importance of CBAM and ASFF in the enhance model

Since accuracy is important in RSBS adults detection tasks, we enhanced YOLOv8m with CBAM + ASFF modules, which outperformed the standard YOLOv8 variants. CBAM enabled the model to focus more effectively on important features, such as the edges and outlines of RBSB adults, while minimizing interference from background noise (Pan and Daoliang, 2022). This selective attention greatly improved the model’s sensitivity to subtle visual cues that are critical for detecting pests in complex agricultural images. ASFF further strengthened the model by allowing it to combine information across different spatial scales (Liu et al., 2019), ensuring robust detection of RBSB adults regardless of their size or orientation.

**Figure 11:**
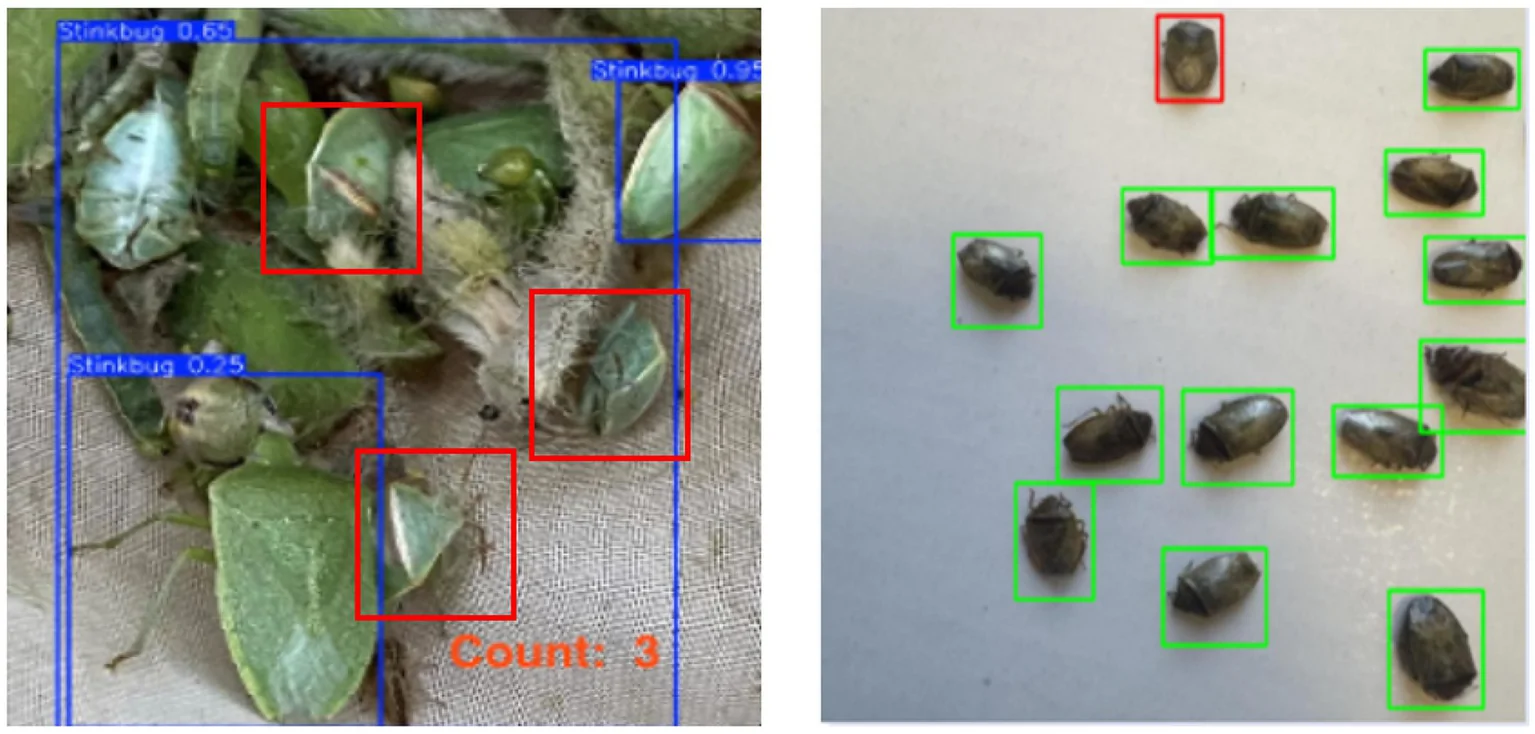
Misdetections of RBSB adults identified by the enhanced model are shown using red bounding boxes.

However, occasional misdetections were likely due to similarities in color, size, or shape that resemble RBSB adults with the background of the images (Figure 11). In addition, inference time increased to 16 milliseconds, but performance still exceeded that of YOLOv8 variants, highlighting the value of these model improvements for more robust detection. Ultimately, the implementation of CBAM and ASFF led to significant gains in detection performance, as reflected by higher precision, recall, and mAP scores compared to the standard models and confirmed with related studies (Pan and Daoliang, 2022; Varghese and Sambath, 2024; Tan et al., 2025). These improvements are particularly important for practical deployment, where accurate and consistent RBSB adults detection can directly support for tracking RBSB population.

### 4.3 Limitations of the enhanced model and model improvement

Despite the strengths of the enhanced YOLOv8m model, our model has some limitations. Detection accuracy is sensitive to the quantity and diversity of images, and overall image quality. (Ranjan et al., 2023; de Melo Lima et al., 2024). Most of the training data included a good range of backgrounds, angles, and lighting conditions; however, field environments can introduce even greater variability, including diverse lighting and differences in sensor resolution that can limit model inference. Although we expanded the dataset with field images from BugGuide and iNaturalist to increase variability, further improvements will require collecting larger and more diverse real-world datasets in uncontrolled settings. This highlights a key area for improvement: increasing the proportion and variability of field images to enhance the model’s robustness and accuracy for detecting and counting RBSB adults.

Furthermore, the enhanced model exhibited only a marginal decrease in recall (≤ 0.4%) compared with YOLOv8s. This slight reduction is unlikely to affect the practical detection of RBSB adults, particularly because the enhanced model achieved a 3% improvement in mAP50 compared to other YOLO variants. This increase in mAP50 reflects a stronger overall detection performance by balancing both precision and recall into a single evaluation metric (Gündüz and Işık, 2023). Nevertheless, the trade-off between accuracy and inference speed may present challenges for deployment on resource-constrained edge devices and should be considered in future research.

### 4.4 Limitations of the web application

The main purpose of our web application is to detect and count RBSB adults, and see the further applicability of this technology into a decision support tool that can also being integrated into unmmaned vehicles for targeted treatment. However, errors can happen when stinkbugs are very small or blend into the background, making them hard to spot. Standard YOLOv8 models often struggle to distinguish pests from backgrounds with similar colors and textures (Vilar-Andreu et al., 2024). For that reason, our application uses the enhanced model for having a higher mAP in detection with lower misdetection error. One of the big limitations of the web application is their functionality depends on internet connectivity and hardware system configuration, which can limit the overall use of end users. Despite the challenges, the web application contributes to understand the capabilities of computer vision models in pest detection, proving foundational alternatives for pest identification. Currently, the web interface counts the RBSB adults in images. Thus, when action threshold is defined by the users, the web application can be upgraded to notify when the threshold exceeds.

### 4.5 Future research

The enhanced model can be deployed in a mobile application to enable real-time detection and counting of RBSB adults using handheld devices. In addition, the framework provides a foundation for creating pest identification tools across different cropping systems. The model can also be trained using images collected by ground-based robotic vehicles for pest scouting, supporting the automation of pest identification, which remains largely manual in many cropping systems.

## 5 Conclusion

During pest monitoring, RBSB adults are typically identified and counted manually, a process that is subjective and prone to error. To automate this task, we developed a training dataset that includes both newly captured images and images sourced from repositories such as iNaturalist and BugGuide. This dataset was used to evaluate multiple computer vision models for detecting and counting RBSB adults. Our best performed model was selected to be enhanced by integrating CBAM and ASFF modules to reduce background noise and improve the ability to capture precise boundaries of RBSB adults, rather than detecting ambiguous objects in images. This resulted in higher detection performance (mAP50) compared to other YOLOv8 variants. The enhanced model demonstrated reliable performance in both detection and counting tasks, offering a practical alternative to traditional visual assessments. The model was implemented in a Streamlit web application, that allows users to upload images for automated pest detection and counting. This application serves as an initial decision support tool that needs further field validation and establishes a foundation for future decision-support systems for pest monitoring.

## Data Availability

The raw data supporting the conclusions of this article will be made available by the authors, without undue reservation.
